# Clay-dye fluorescent hybrid material for the detection of neurotransmitter secretion from cells

**DOI:** 10.1039/d5nr03358f

**Published:** 2025-10-28

**Authors:** Giorgia Giovannini, Ada De Luigi, Luciano F. Boesel, Pierre Picchetti, Frank Biedermann

**Affiliations:** a Department of Biochemistry and Molecular Pharmacology, Mario Negri Institute for Pharmacological Research IRCCS Via Mario Negri 2 Milano Italy giorgia.giovannini@marionegri.it; b Empa, Swiss Federal Laboratories for Materials Science and Technology, Laboratory for Biomimetic Membranes and Textiles Lerchenfeldstrasse 5 St. Gallen 9014 Switzerland; c Institute of Nanotechnology (INT) Karlsruhe Institute of Technology (KIT) Kaiserstrasse 12 76131 Karlsruhe Germany pierre.picchetti@kit.edu frank.biedermann@kit.edu

## Abstract

We present a clay-dye hybrid material (MDAP@MMT) for the rapid and selective fluorescence-based detection of catecholamine neurotransmitters. The probe is formed by adsorption of the fluorescent dye 2,7-dimethyldiazapyrenium (MDAP) onto montmorillonite clay (MMT), resulting in partial fluorescence quenching that is further suppressed upon dopamine binding. In aqueous media, the probe responds within one minute and follows a 1 : 1 binding model with dopamine, yielding an average binding constant of *K*_a_ = (8.3 ± 1.1) × 10^4^ M^−1^ and a detection limit of 12 µM. Embedding the probe in agarose hydrogels enhances its stability and enables operation in complex media such as cell culture medium. As proof of concept, the system successfully detected dopamine released from neuroblastoma cells upon stimulation, demonstrating its potential for *in vitro* neurochemical sensing. The simplicity, responsiveness, and low-cost nature of MDAP@MMT underscore its potential for integration into future sensing platforms aimed at biomedical or diagnostic applications.

## Abstract 2

We present a clay-dye hybrid material (MDAP@MMT) for the rapid and selective fluorescence-based detection of catecholamine neurotransmitters.

## Graphical abstract

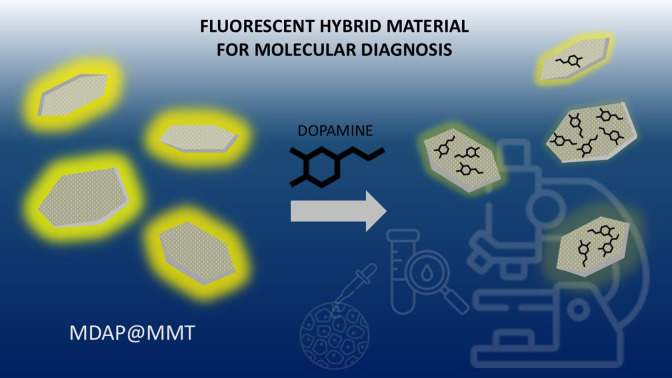

## Introduction

Neurotransmitters (NT) are small molecules that control neural communication and thus regulate human activities such as behavior, memory, and cognition. Within the central nervous system (CNS), they also represent biomarkers for certain neurodegenerative diseases, which pose a major challenge for an ageing global population, with the prediction that one in six people over the age of 65 will be affected by such a debilitating condition by 2050.^1^ The tightly controlled spatial and temporal release of NTs is essential for normal brain function, while dysregulation is implicated in a wide spectrum of disorders, including Parkinson's disease, schizophrenia, depression, ADHD, and neurodegenerative conditions.^2,3^ In particular, dopamine (DA) depletion in the CNS is a hallmark of Parkinson's disease, with levels dropping to below 3–5% of normal in late-stage patients.^4–6^ Such a drop in DA levels eventually leads to motor symptoms (*e.g.* tremor and rigidity), which are the first visible signal that occurs only at advanced stages of the disease.

Early and decentralized detection of DA in physiological fluids such as cerebrospinal fluid (CSF) or blood could offer valuable insights for clinical diagnostics, therapeutic monitoring, and basic neuropharmacological research.^7^ In this context, the ability to monitor NTs may contribute to a better understanding of neural signaling and potentially support the early recognition of neurological disorders.^8^ Microdialysis enables the quantification of NTs by inserting semipermeable membrane-based probes in the brain tissue.^9^ This technique allows monitoring the level of specific compounds in defined areas of the tissue, but it is invasive, requires trained personnel and costly instrumentation.^10^ Traditional approaches for NTs quantification, such as fluorescence or electrochemical detection, capillary electrophoresis, liquid chromatography and mass spectrometry, provide high sensitivity but are often constrained by labor-intensive sample preparation, high instrumentation costs, or limited portability. In contrast, more recent sensing strategies exploit nanomaterials, optical transduction mechanisms, and molecular recognition elements to develop rapid and miniaturized alternatives to conventional methods.^11^ Examples include electrochemical methods,^12–14^ optically based techniques,^15,16^ nanozymes,^17^ and nanomaterials.^18,19^ Recent advancements have opened promising avenues using hybrid materials, combining organic molecules and inorganic substrates to utilize enhanced optical and electronic properties for NTs sensing. Among such materials, clay minerals, particularly montmorillonite (MMT), stand out due to their natural abundance, biocompatibility, high surface-to-volume ratio, and intrinsic cation exchange capacity.^20^ The intriguing photophysical behavior of dyes interacting with MMT and clays in general has been extensively studied in the last decades.^21–23^ Clay-dye hybrid systems, in particular, represent a unique class of optical sensor materials. Ikeda *et al.* developed an innovative MMT-based hydrogel hybrid, in which a cationic coumarin dye displayed distinct photophysical changes upon interaction with polyamines, enabling highly sensitive biomarker detection.^24^ Similarly, Zhu and colleagues constructed a visible-light-triggered colorimetric probe by integrating porphyrin-functionalized TiO_2_ nanoparticles onto MMT, exploiting its enhanced peroxidase-like catalytic activity for selective glutathione detection down to nanomolar concentrations.^25^ Hybrid systems were also prepared by modifying MMT with metals, achieving optical sensing platforms enabling the label-free and SERS detection of tryptophan.^26^ In parallel, porous aluminosilicate-based materials, such as nanozeolite receptors have demonstrated extraordinary specificity and affinity for NTs host–guest-type interactions, advancing the understanding and capabilities in biomolecular sensing.^27,28^ In another example, Zang *et al.* developed a ratiometric fluorescence probe based on zeolitic imidazolate framework-8 encapsulating gold nanoclusters and graphene quantum dots for sensitive and selective detection of dipicolinic acid, an anthrax biomarker.^29^

Herein, we present a straightforward optical sensing strategy in which the dicationic dye 2,7-dimethyldiazapyrenium (MDAP) is adsorbed on MMT. The resulting hybrid (MDAP@MMT) exhibits a strong, concentration-dependent fluorescence quenching by aromatic NTs and, when embedded in agarose, forms solid films that allow user-friendly *in vitro* assays with mid-micromolar detection limits, appropriate for pharmacological screening, cell culture studies, or model biofluids.

## Results and discussion

**Fig. 1:**
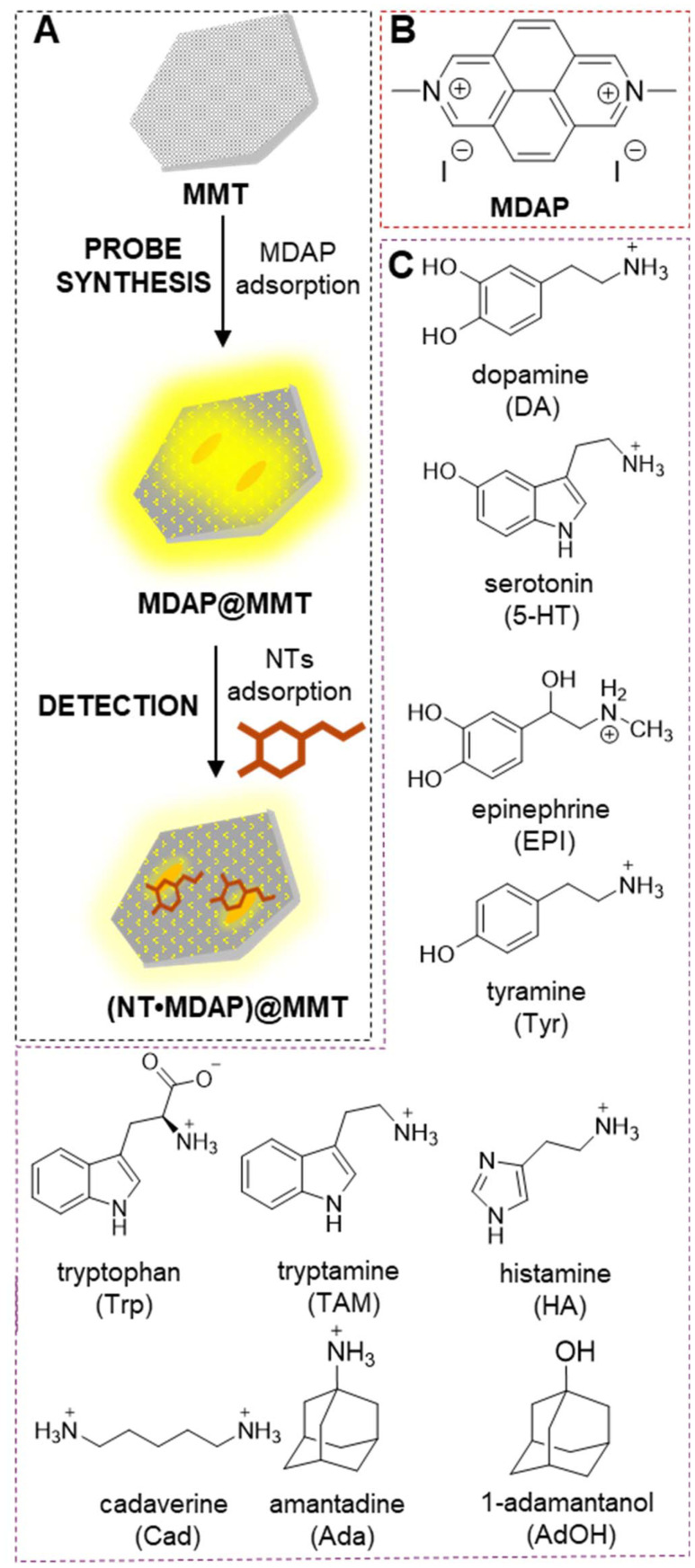
(A) Schematic illustration of the preparation of the MDAP-MMT probe *via* simple mixing of the dye and clay suspension, followed by analyte detection. (B and C) Chemical structures of the fluorescent dye MDAP (B) and selected analytes such as NTs and biogenic amines investigated in this study (C).

The sensing probe developed in this study for NT detection was designed by exploiting the tunable optical properties of clay-dye hybrid materials. It consists of: (i) the reporter dye MDAP,^27^ whose fluorescence can be modulated through physicochemical interactions involving electron transfer processes;^28^ and (ii) MMT, a naturally occurring layered clay mineral known for its high adsorption capacity, large specific surface area, and swelling behavior.^30^ The preparation of the hybrid dye-clay material *via* simple mixing of a clay suspension with an MDAP solution is schematically illustrated in Fig. 1.

**Fig. 2:**
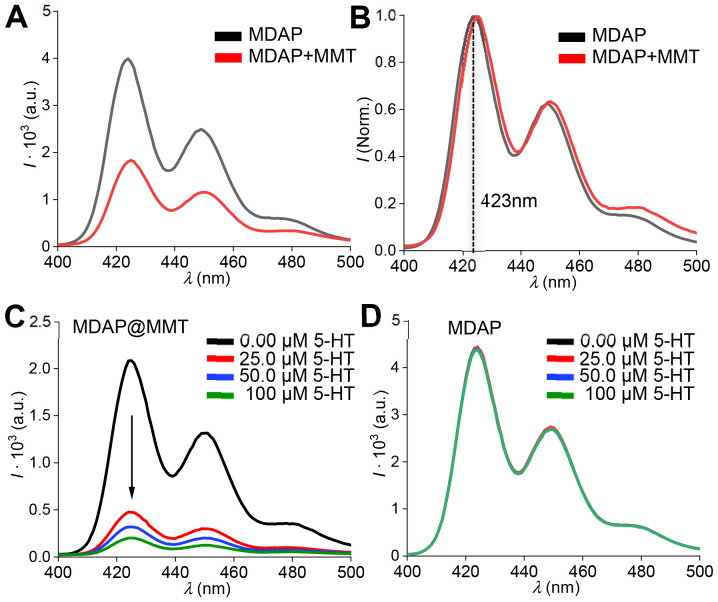
(A) Fluorescence spectra of MDAP (50 µM) in the absence (black) and presence (red) of MMT (0.01 wt%) after 15 minutes of incubation (*λ*_ex_ = 339 nm). Adsorption onto MMT leads to a ∼50% reduction in fluorescence intensity. The 15 minute incubation ensures equilibrium conditions are reached. (B) Normalized spectra. (C) Fluorescence quenching of MDAP@MMT in response to increasing concentrations of serotonin (SE, 0–100 µM). (D) Control experiment showing that free MDAP in solution exhibits no quenching under identical SE concentrations, highlighting the essential role of MMT in the sensing mechanism.

As shown in Fig. 2A, adsorption of MDAP onto the MMT layers resulted in significant fluorescence quenching, reducing the emission intensity by approximately half, while the spectral shape remained unchanged (Fig. 2B). The observed adsorption of MDAP onto the MMT surface can primarily be attributed to electrostatic interactions, owing to the dicationic nature of MDAP and the negatively charged sites present on MMT. Additionally, weaker, non-specific interactions such as van der Waals forces or hydrophobic interactions could contribute to stabilizing this binding. The partial fluorescence quenching observed upon adsorption likely results from processes that occur after the fluorophore binds to the clay surface. Possible mechanisms include surface-induced energy transfer phenomena, such as Photoinduced Electron Transfer (PET) or Surface Energy Quenching (SEQ), facilitated by proximity to the clay mineral surface.^23^

MDAP was chosen because it forms direct noncovalent contacts with NTs, enabling PET that leads to variation of the optical properties, typically emission quenching.^31^ As confirmation, the signal of the so-achieved hybrid probe decreased from 2.00 × 10^3^ to 0.50 × 10^3^ a.u. and 0.25 × 10^3^ a.u. when treated with 25 and 100 µM of the NTs serotonin (5-HT), indicating a non-linear response typical for saturation-type binding (Fig. 2C). On the contrary, the emission intensity was not altered when an aqueous solution of MDAP was treated with 5-HT (0–100 µM), highlighting the role of MMT in the here proposed sensing mechanism (Fig. 2D). Owing to its large surface area and inherent surface charge, MMT provided a platform for the co-adsorption of MDAP and 5-HT. This spatial confinement facilitated their mutual interaction and enabled photophysical processes, such as PET, to occur at micromolar concentrations of dye and analyte, whereas in bulk solution, such interactions are observed only at millimolar levels between these species.^27^

Titration experiments were conducted to optimize the adsorption of MDAP onto MMT (Fig. S1). When MDAP solutions at 50 µM and 25 µM were titrated with increasing concentrations of MMT (Fig. S1A–C), fluorescence quenching reached a saturation point at MDAP : MMT ratios of approximately 2500 (*i.e.*, 25 µM : 0.01 wt% and 50 µM : 0.02 wt%). Conversely, titrating a constant MMT concentration of 0.05 wt% with serial dilutions of MDAP revealed that signal stabilization occurs at 50 µM MDAP (Fig. S1D and E). Pristine MMT and MDAP@MMT characterized with Scanning Electron Microscopy (SEM) and Electrophoretic Light Scattering (ELS). The results showed that the adsorption of MDAP did not disrupt the layered structure of the particles (Fig. S2A), whereas the surface charge became slightly more negative, from −22.57 ± 0.42 mV to −31.37 ± 1.68 mV (Fig. S2B). The observed increase in the negative zeta potential of MMT upon adsorption of positively charged molecules such as MDAP can be explained by the fact that *ζ* reflects the electrical potential at the slipping plane rather than at the actual surface, where charge inversion may occur. When a large number of cations are strongly adsorbed onto negatively charged surfaces, they can overcompensate the surface charge and attract an excess of anions into the slipping plane.^32^ This redistribution results in a more negative zeta potential, despite the adsorption of positively charged molecules. Several theoretical and experimental studies have shown that such charge inversion and over-compensation phenomena at the slipping plane originate from ion–ion correlations and multivalent counterion effects.^33,34^

The influence of the MDAP-to-MMT ratio on neurotransmitter responsiveness was also evaluated using epinephrine (EPI) as an additional model analyte. These measurements demonstrated that the signal change in the MDAP@MMT composite correlates with the MDAP concentration: higher MDAP levels yielded greater sensitivity to EPI, with response saturation occurring at a 1 : 1 ratio of MDAP to analyte (Fig. S3A). In contrast, varying the MMT concentration had no discernible effect on detection efficiency (Fig. S3B), indicating that MMT primarily serves as a passive adsorption matrix that promotes the spatial colocalization of MDAP and analyte, rather than directly participating as an independent EPI binding station. Based on these findings, a ratio of 50 µM MDAP to 0.05 wt% MMT was selected for probe preparation. This formulation is hereafter referred to as MDAP@MMT. The long-term stability of the probe was confirmed *via* dialysis experiments, which showed that MDAP remained adsorbed on MMT over a period of 7 days, with no detectable release into the supernatant and no measurable change in fluorescence intensity (Fig. S4).

**Fig. 3:**
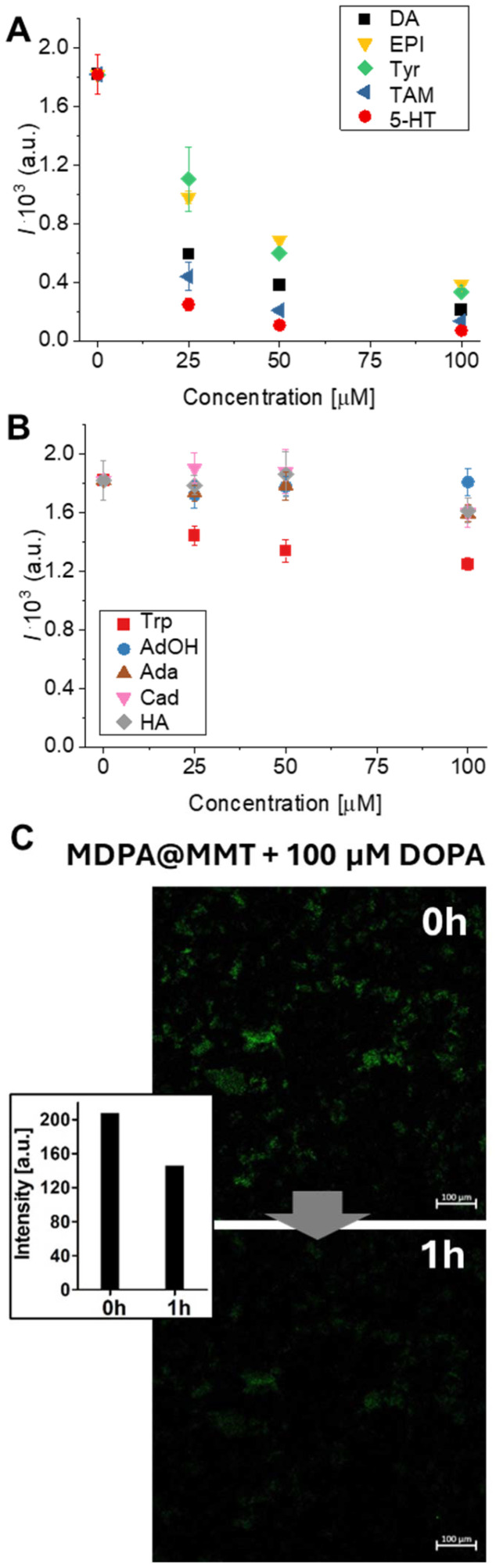
Plots of fluorescence emission intensity at 425 nm (*λ*_ex_ = 339 nm) as a function of NT's concentration, measured using the MDAP@MMT sensing probe. (A) Catecholamine- and amine-derived NTs (*i.e.* DA, EPI, 5-HT, Tyr, and TAM) induce a concentration-dependent decrease in fluorescence intensity, indicative of electron transfer-based quenching. (B) In contrast, structurally and electronically dissimilar compounds (*i.e.*, Trp, Ada, AdOH, HA and Cad) produce significantly lower fluorescence change, confirming the selectivity of the probe. Data represents mean values ± standard deviation (*n* = 3). (C) Confocal fluorescence microscopy image showing the quenching effect observed for MDAP@MMT after treatment with 100 µM DA. Inset: quantification of the fluorescent intensity using ImageJ. After 1 h of treatment, the probe showed a decrease in the fluorescent intensity to 146 a.u. compared to the initial value 208 a.u.

Once the MDAP@MMT probe was characterized, its sensing performance was assessed in terms of sensitivity and selectivity toward NTs and structurally related compounds. As shown in Fig. S5, fluorescence intensity decreased significantly upon incubation with NTs such as DA, 5-HT, EPI, and their biosynthetic precursors Tyr and TAM (i–v). In contrast, no appreciable fluorescence change was observed for compounds such as Trp, Ada, AdOH, Cad and HA (vi–x). Kinetic analyses demonstrated that fluorescence quenching occurred rapidly, within 60 seconds, for responsive analytes (Fig. S6i–v), while unresponsive compounds produced no time-dependent signal changes (Fig. S6vi–x). These findings indicate that the quenching is not due to nonspecific effects but rather results from defined physicochemical interactions such as PET between the MDAP@MMT hybrid and select NTs. The selectivity of the MDAP@MMT probe can be explained by the electronic properties of the analytes, particularly their ability to act as electron donors. As shown in Fig. 1C, DA, 5-HT, EPI, Tyr, and TAM all feature electron-rich aromatic moieties, such as catechol, phenol, or indole rings, which facilitate PET to the excited-state MDAP fluorophore (Fig. 3A). This interpretation is consistent with previous reports on MDAP and related viologens, where photoinduced electron transfer with aromatic electron-rich guests has been established in CB8- and zeolite-based host–guest systems.^35,36^ Statistical analysis (ANOVA one-way test, *n* = 3, *α* 0.05, *post hoc* Tukey's test) indicated that the signal variation was significantly different for all compounds at the different concentrations, reaching a saturation for TAM and 5-HT between 50 and 100 μM (Fig. S7A). These donor–acceptor interactions effectively quench the fluorescence of MDAP by modulating its excited-state electronic structure.^37–39^ The extent of quenching correlates with the electron-donating capacity and structural compatibility of each analyte, all of which promote noncovalent proximity to MDAP through interactions supported by the MMT surface. In contrast, less pronounced fluorescence changes were recorded in response to compounds such as Trp, HA, Ada, AdOH, and Cad (Fig. 1C and 3B). Trp occurs predominantly in zwitterionic form at physiological pH, which limits its electrostatic interaction with the negatively charged MMT surface. As a result, this compound likely exhibits reduced binding and fails to engage in the electronic interactions necessary to induce quenching. Moreover, aliphatic or electron-poor aromatic structures such as Ada, AdOH, Cad, and HA do not provide the conjugated systems required for PET with MDAP.^40^ Although aliphatic amines such as HA, Cad, and Ada likely interact electrostatically with the negatively charged MMT surface, they are too electron-poor to induce PET with MDAP, consistent with the absence of significant spectral changes. The limit of detection (LOD) for catecholamine-type NTs DA and EPI was determined to be below 20 µM (Fig. S8), demonstrating the probe's sensitivity toward physiologically relevant NT concentrations. Statistical analysis (ANOVA one-way test, *n* = 3, *α* 0.05, *post hoc* Tukey's test) confirmed the limited variation of MDAP@MMT signal upon treatment with these compounds indicating a significant decrease only with Trp at 25 μM and Ada and HA at 100 μM (Fig. S7B). Titration data fitted to a 1 : 1 direct binding assay (DBA) model confirmed a high-affinity interaction between MDAP@MMT and DA, yielding *K*_a_ = (8.3 ± 1.1) × 10^4^ M^−1^ (Fig. S9). To complement fluorescence spectroscopy, the probe's response was further evaluated by confocal fluorescence microscopy (Fig. 3C). Upon incubation with 100 µM DA for 1 hour, a clear reduction in fluorescence intensity was observed, confirming the probe's suitability for integration into imaging-based sensing platforms. Confocal fluorescence microscopy revealed a homogeneous distribution of MDAP across the MMT surface, consistent with adsorption at the basal planes of the clay particles. Such adsorption is primarily driven by electrostatic interactions with the negatively charged siloxane layers of MMT, with cationic moieties of MDAP anchoring efficiently to these sites, often reinforced by hydrogen bonding with surface oxygen atoms. As we have previously shown for xanthene dyes and coumarins,^23^ adsorption at the basal planes of MMT can induce pronounced photophysical effects arising from H-aggregation, π–π interactions, and hydrogen bonding to surface oxygen atoms. Confocal fluorescence microscopy likewise revealed a homogeneous distribution of MDAP across the MMT surface, consistent with electrostatic adsorption at the negatively charged siloxane layers. In contrast to the behavior of neutral or singly charged dyes, the dicationic MDAP, being less prone to stacking interactions, exhibited only a slight redshift in emission wavelength and a modest decrease in emission intensity upon binding to MMT. Despite the low concentration of NTs used, Fourier Transform Infrared (FT-IR) Spectroscopy was useful to confirm the adsorption of DA on MDAP@MMT. After treatment with 100 μM of DA, the probe was isolated by centrifugation and analyzed. The spectrum of the dried samples shows the peaks of N–H (primary amine) and O–H stretching (3340–3200 cm^−1^ region), and the stretching of aromatic C–H at 3040 cm^−1^, also observed in the spectra of DA and absent in MDAP@MMT (Fig. S10).

Flocculation of the MDAP@MMT probe was observed when stored in aqueous suspension over time. Although the aggregates could be readily redispersed by brief shaking, such instability risks compromising reproducibility, particularly in dynamic or complex biological settings such as cell culture media or clinical specimens. To enhance the stability and robustness of the sensing performance, MDAP@MMT was embedded within hydrogel scaffolds designed to balance mechanical integrity and analyte diffusivity. Three biocompatible matrices were tested: agarose (1% w/v), gelatin (5% w/v), and calcium alginate (2% w/v alginate crosslinked with 1% w/v CaCl_2_). No detectable leakage of MDAP@MMT was observed from any of the matrices, likely due to the submicron size of MMT and its hydroxyl-functionalized surface, which promotes favorable physicochemical interactions with the hydrogel network. However, only agarose-embedded MDAP@MMT retained sensitivity toward DA. Probes embedded in gelatin and alginate displayed altered response behavior, presumably due to the presence of amine and carboxyl groups in the polymer matrices, which may interfere with analyte binding or probe–analyte interactions (Fig. S11A).

**Fig. 4:**
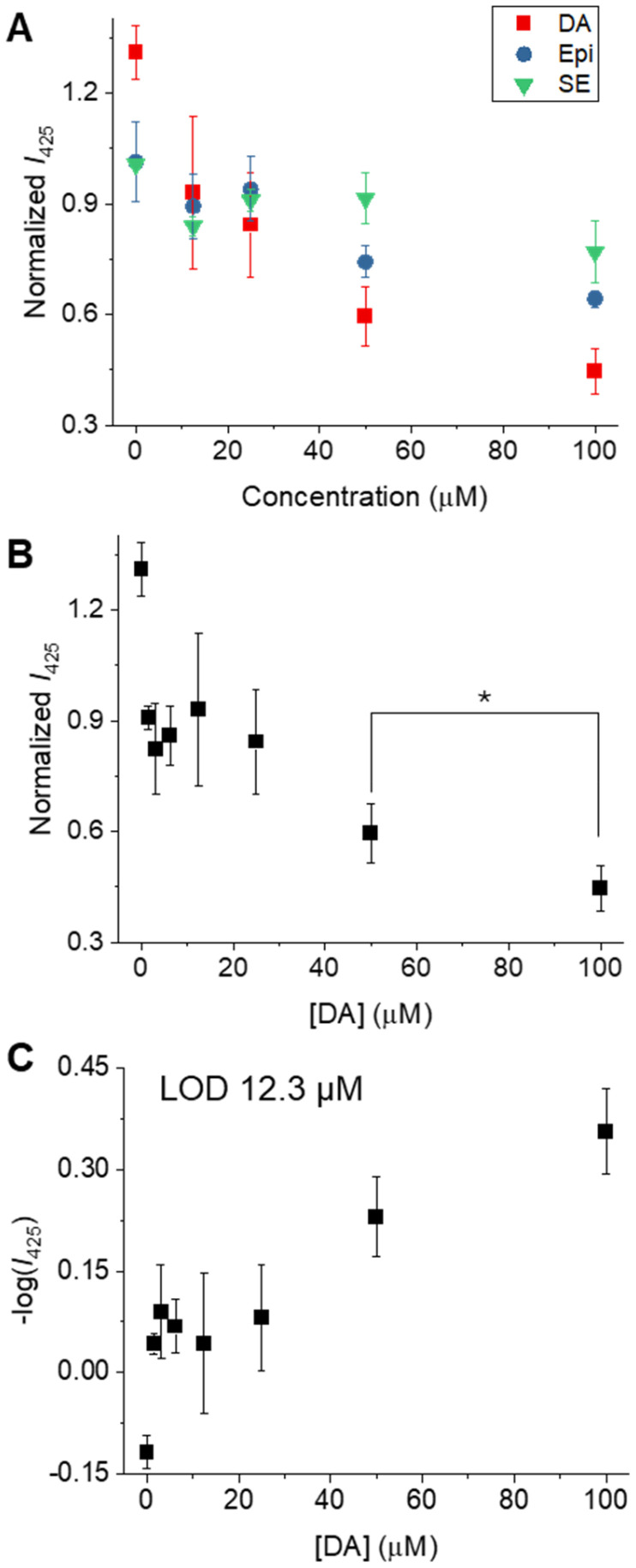
Performance of MDAP@MMT-loaded agarose films for detecting catecholamine NTs in DMEM buffer. (A) Fluorescence response (normalized I425, *λ*_ex_ = 339 nm) of the probe after treatment with DA, 5-HT, and EPI in the concentration range of 0–100 µM. The probe shows highest sensitivity and selectivity toward DA. (B) Concentration-dependent fluorescence quenching curve for DA, showing a clear and linear decrease in signal. *Sample means are statistically different (two-tailed *t*-test, *n* = 3, *p* < 0.05, *p* = 0.0396). (C) Plot of −log(*I*_425_) *vs.* DA concentration, used for LOD calculation based on the linear regression in the range 0–50 µM. The LOD was calculated as LOD = 3.3*σ*/*S* and found to be 12.3 µM. Data are shown as mean ± standard deviation (*n* = 3). Fitting parameters: DA − standard error of regression (*S*) = 1.9, *R*^2^ = 0.87; EPI − *S* = 0.9, *R*^2^ = 0.91; SE − *S* = 2.9, *R*^2^ = 0.57.

Incorporation into agarose effectively stabilized the probe's fluorescence signal over time, as demonstrated by comparative measurements in Dulbecco's Modified Eagle Medium (DMEM) containing 10% Fetal Bovin Serum (FBS), where the agarose-loaded probe outperformed the free suspension (Fig. S11B). There is a notable difference in response time between the free MDAP@MMT probe and its hydrogel-embedded counterpart. While the free suspension stabilizes its fluorescence signal within 60 seconds upon dopamine addition, the agarose-embedded version requires approximately 90 minutes to reach equilibrium (Fig. S11C). This delay can be attributed to the diffusion barrier imposed by the hydrogel matrix, which slows the transport of analytes to the sensing interface. Nevertheless, this increase in response time represents a worthwhile compromise, given the enhanced physical stability and suitability of the embedded format for applications involving complex media or prolonged measurement conditions (Fig. S11D). Moreover, beyond providing a chemical anchor for the clay particles, the hydrogel can also function as a size-exclusion barrier, preventing larger components of human samples, such as proteins, from interacting with the probe. The suitability of the MDAP@MMT-based probe for detecting NTs under biologically relevant conditions was assessed using agarose-embedded films. These were tested against DA, 5-HT, and EPI in DMEM (Fig. 4A). To ensure consistent baseline fluorescence, the films were pre-incubated in medium for one hour prior to analyte exposure. Afterward, the gels were treated with NTs at concentrations ranging from 0–100 µM, and fluorescence intensity was measured two hours post-incubation. The films demonstrated a strong responsiveness toward DA, exhibiting a near-linear decline in emission intensity. At 100 µM DA, fluorescence dropped to approximately 45% of the initial signal (Fig. 4B). For quantification, the minus log of the fluorescence intensity was plotted against DA concentration (0–50 µM), yielding a linear relationship (*R*^2^ = 0.95, slope = 0.0065) well-suited for analysis of quenching-type sensing mechanisms. The LOD was derived from the regression parameters using the standard formula LOD = 3.3*σ*/*S*, where *σ* is the standard deviation of the blank and *S* is the slope of the calibration curve, an approach commonly applied in fluorescence-based sensing.^41,42^ The calculated LOD for DA was 12.3 µM, which closely mirrors the 11.0 µM determined for the free MDAP@MMT probe in aqueous suspension. In contrast, the sensitivity of the hydrogel-embedded probe toward 5-HT was markedly reduced, with the LOD rising from 2.80 µM (free probe) to 17.0 µM loaded in gel. This diminished response may be attributed to interactions between 5-HT and the agarose matrix, potentially hindering effective probe-analyte interaction.^43^ We also tested the responsiveness of MDAP@MMT toward L-DOPA, a drug used in dopaminergic therapy. While modest fluorescence quenching was observed, the signal exhibited high variability (Fig. S12), consistent with weak and reversible adsorption. This is attributed to L-DOPA's zwitterionic form at physiological pH, in contrast to dopamine's cationic state. Considering that extracellular L-DOPA concentrations are very low in the absence of drug administration and that its interaction with the probe is weaker than with DA, the probe's performance under normal physiological conditions is expected to remain uncompromised.^44^

**Fig. 5:**
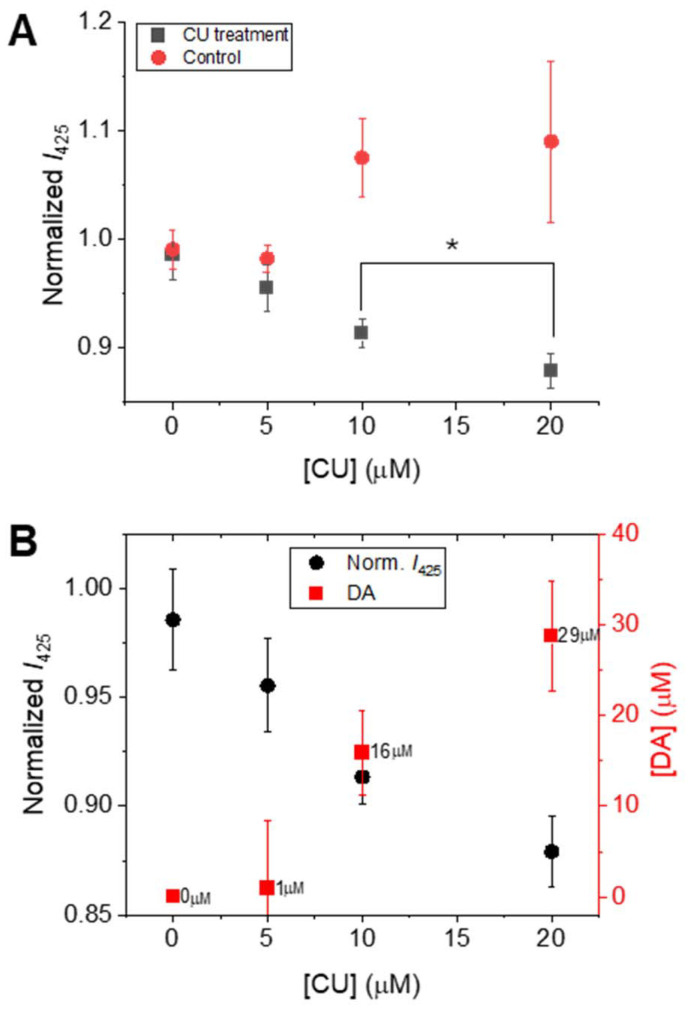
Evaluation of DA production from dopaminergic neurons upon CU stimulation using MDAP@MMT-loaded agarose films. (A) Normalized fluorescence intensity of the probe after incubation with either cell supernatants from CU-treated dopaminergic neurons (gray squares) or with CU alone (red circles) at concentrations of 0, 5, 10, and 20 µM (*λ*_ex_ = 339 nm). A decrease in signal is observed in the presence of DA-containing supernatants, while a slight increase is seen upon direct exposure to CU alone. *Sample means are statistically different (two-tailed *t*-test, *n* = 3, *p* < 0.05, *p* = 0.0219). (B) Quantification of DA concentration (red squares, right axis) released in response to increasing CU levels, calculated from the corresponding calibration curve. The decrease in normalized fluorescence intensity (black circles, left axis) correlates with the rise in DA levels. Data represent mean ± standard deviation (*n* = 3).

As a proof of concept, the MDAP@MMT probe was employed to evaluate the capacity of curcumin (CU) to stimulate DA release from dopaminergic neurons. For this purpose, 10^5^ cells per well were seeded in a 24-well plate and treated with increasing concentrations of CU (0, 5, 10, and 20 µM) for 18 hours. CU is known to enhance DA biosynthesis by activating signaling pathways in dopaminergic cells.^45,46^ The cells were incubated with CU in Hank's Balanced Salt Solution (HBSS), following previously reported protocols.^47^ After incubation, the culture medium was collected, centrifuged at 13 000 rpm for 5 minutes, and 100 µL of the supernatant was transferred to 96-well plates containing MDAP@MMT-loaded agarose films. The films were incubated with the supernatant for 2 hours, after which the fluorescence intensity was measured. A linear decrease in fluorescence intensity was observed with increasing CU concentration (Fig. 5), reflecting a dose-dependent release of DA by the dopaminergic cells. As a control, the MDAP@MMT probe was directly incubated with CU in the absence of cells. Here, a slight increase in fluorescence was observed, which may be due to the interaction of CU with MMT,^48^ thereby confirming that the signal decrease in the test samples originated from DA production rather than direct probe-compound interaction. Quantification of DA was achieved by referencing the corresponding calibration curve (Fig. S13), demonstrating the probe's applicability for *in vitro* monitoring of neurotransmitter release and therapeutic modulation.

## Experimental

### Materials

Montmorillonite Dellite® LVF (Cation Exchange Capacity 105 meq per 100 g) was donated by Laviosa Chimica Materia (Livorno, Italy). Agarose BioReagent, for molecular biology, alginic acid sodium salt from brown algae, calcium chloride, gelatin from bovine skin, dopamine hydrochloride, serotonin, (−)-epinephrine, 4-(2-hydroxyethyl)piperazine-1-ethanesulfonic acid (HEPES) were purchased from Sigma-Aldrich (Buchs, Switzerland). l-Tryptophan, histamine, and tyramine were purchased from Fluka. 2,7-Dimethyldiazapyrenium Diodide (MDAP) was synthesized according to literature procedures.^27^ Curcumin was purchased from Merck Life Science (Milan, Italy). Dulbecco's Modified Eagle's medium (DMEM) with and without phenol red, Fetal Bovine Serum (FBS), trypsin-EDTA, Hanks’ Balanced Salt Solution (HBSS) were purchased from Gibco Thermo Fisher Scientific (Monza, Italy).

### Preparation of MDAP@MMT

MMT was dispersed in DI water at 1% (wt%) concentration and stirred for three days at room temperature. The suspension was sonicated (for 30 min, 50 W, frequency 35 kHz) and centrifuged (at 5765 G-force for 10 min) three times to remove metal traces. The supernatant was collected, reaching the 1 wt% MMT stock solution. An aqueous solution of MDAP was mixed with an aqueous suspension of MMT and stirred for 15 minutes to facilitate dye adsorption. The final sensing probe was prepared by combining MDAP and MMT at final concentrations of 50 µM and 0.05% (wt%), respectively. This ratio was selected based on titration experiments in which MDAP (0–50 µM) was incubated with varying concentrations of MMT (0–0.07 wt%), allowing identification of optimal conditions for probe formation.

### Characterization of the probe

Pristine MMT and the MDAP@MMT were characterized in terms of morphology and surface charge. SEM images were acquired using a Hitachi S-4800 scanning electron microscope at an acceleration voltage of 20 kV and magnifications from 10 k to 20 k. The samples were dropped on conductive carbon tape and sputter-coated with 7 nm of Au/Pd alloy. *ζ*-potential was measured by Zetasizer NanoZS (Malvern Instruments Ltd, Malvern, UK) through a HeNe laser of 633 nm with a backscatter angle of 90°, using disposable folded capillary cell (DTS1070, Malvern Instruments Ltd, Malvern, UK). FT-IR spectroscopy was performed using a Varian 640-IR spectrometer. The spectra were recorded in the spectral range of 4000–450 cm^−1^ with a sample scan of 64 and a resolution of 4 cm^−1^.

### Spectroscopic response of MDAP@MMT dispersions to analytes

A dispersion of MDAP@MMT was prepared as described in the section Preparation of MDAP@MMT, with the difference that the 1 wt% MMT dispersion was subsequently diluted and mixed with MDAP in 10 mM HEPES buffer at pH 7, resulting in final concentrations of 50 µM dye and 0.05 wt% clay. The solution was then allowed to mix for 15 minutes before the analytes (0–100 µM) were added, and the emission spectra were recorded at an excitation wavelength of *λ*_ex_ = 339 nm, with emission collected in the range of *λ*_em_ = 400–500 nm.

### Titration of MDAP@MMT with DA and 1 : 1 direct-binding model (DBA)

An aqueous suspension of MDAP@MMT (0.05 wt% MMT, 50 µM MDAP; 2 mL in a 4-side-clear quartz cuvette) was titrated with a 2.912 mM dopamine stock solution (0–112 µM final concentration) using a JASCO FP-8300 spectrofluorometer equipped with an FP-8300 titration set-up (STR-812 stirrer + ATS-827 automatic titrator, 2 µL per step, 40 injections). Emission intensity was recorded after each addition at *λ*_ex_ = 339 nm (bandwidth = 1 nm) and *λ*_em_ = 425 nm (bandwidth = 2.5 nm); response time = 1 s; Xe lamp source; medium sensitivity setting. The raw traces were normalized to the initial intensity and fitted with the open-source FittingApp (version 0.4.4; https://github.com/ahmadomira/fitting-app-releases) using a 1 : 1 direct-binding model (DBA). For each replicate the parameters *K*_a_, *I*_o_, *I*_MDAP@MMT_ and *I*_(DA·MDAP)@MMT_ were optimized; goodness-of-fit was assessed by the root-mean-square error (RMSE) and the coefficient of determination (*R*^2^). All fits converged to *K*_a_ = 7.0 × 10^4^ M^−1^–10.0 × 10^4^ M^−1^ with excellent statistics (RMSE ≤ 0.012, *R*^2^ ≥ 0.994). Averaging the replicas gives (mean ± SD, *n* = 5): *K*_a_ = (8.28 ± 1.08) × 10^4^ M^−1^, *I*_o_ = (5.57 ± 7.46) × 10^−5^, *I*_MDAP@MMT_ = (1.94 ± 0.13) × 10^4^ M^−1^, *I*_(DA·MDAP)@MMT_ = (8.22 ± 0.35) × 10^3^ M^−1^.

### Preparation of MDAP@MMT hydrogels

MDAP@MMT was prepared by mixing 1 mM of MDAP and 1 wt% of MMT. Agarose 1% (w/v) and gelatin 5% (w/v) were solubilized in hot DI water. MDAP@MMT was added to the hydrogel solutions with a dilution factor of 20. The mixtures were left to gel at room temperature. Alginate 2% (w/v) was solubilized in DI water. Then, the probe was added with a dilution factor of 20 and the hydrogel was formed by nebulization of calcium chloride solution (1% w/v). The films were formed in Petri dishes, reaching an approximate thickness of 1 mm.

### Evaluation of the responsiveness of MDAP@MMT agarose films

The films were cut and plated in a 96-well plate with a flat bottom. The films were incubated for 2 hours with 100 µL of buffer solution (HEPES or DMEM – no phenol red, +10% FBS) to equilibrate the fluorescent signal. The liquid was then replaced with 100 µL of the tested solution. The fluorescent signal was measured with a microplate reader (BioTek Synergy H1).

### *In vitro* assay

Human neuroblastoma SH-SY5Y cell line was chosen as neuronal dopaminergic model (ATCC-CRL-2266, LGC Standards). Cells were maintained in complete medium (DMEM high glucose concentration with phenol red, 2 mM Glutamine, 1% Pen/Strep + 10% FBS, pH 7.2–7.4), detached by trypsin-EDTA (0.25%), seeded (10^5^ per ml per well) in 24-well plate in complete medium, and incubated for 24 hours (37 °C, in a humidified 5% CO_2_ atmosphere). The medium was replaced with curcumin (0, 5, 10, and 20 µM, in HBSS 1×, pH 6.7–7.8) for 18 hours. After the treatment, the medium was collected and centrifuged at 13 000 rpm for 5 min. 100 µL of the supernatant was incubated with the MDAP@MMT-loaded agarose gel, previously plated and equilibrated with HBSS medium. The fluorescent signal of the probe was measured after 2 hours.

### Statistical analysis

Statistical analysis was accomplished using GraphPad Prism software. To compare different samples, one-way ANOVA with a *post hoc* test was used to define the relationship between groups (*α* 0.05, *post hoc* Tukey test). The variation of the measured values of MDAP@MMT probe for dopamine was statistically analyzed using a two-tailed *t*-test (*p* > 0.05, confidence interval 95%).

## Conclusions

In this work, we introduce a fluorescence-based hybrid probe (MDAP@MMT) for the rapid detection of catecholamine-type NTs, achieving selective signal quenching within one minute. The probe can be readily incorporated into agarose hydrogel films, which enhances its stability and preserves sensing performance in complex media, although a longer equilibration time was needed. Notably, the agarose-embedded system displayed high selectivity toward DA over other zwitterionic amino acids or aliphatic cationic or non-charged compounds, with a LOD of 12.3 µM. To demonstrate its utility in biomedical contexts, we validated the use of MDAP@MMT films in monitoring DA release from dopaminergic neurons upon treatment with curcumin, confirming the system's suitability as an *in vitro* assay for assessing neuroactive compounds. DA is a widely studied sensing target, as it can be addressed by electrochemical and chemical approaches exploiting its reducing properties, as well as by recognition of its catechol (diol) moiety through boronic acids. Representative literature reports with their detection limits are summarized in Table S1. It should be emphasized that detection systems sensitive in the micromolar range, such as MDAP@MMT, are particularly efficient at physiologically relevant levels since they are not affected by signal saturation and are therefore particularly suitable, as demonstrated here, for monitoring modulatory effects (*e.g.*, curcumin) on dopamine release from cells. The MDAP@MMT probe combines low cost, straightforward fabrication, and optical responsiveness, making it well-suited for the detection of biologically relevant catecholamines. Its applicability in both suspension and hydrogel formats enables flexible use in bioanalytical settings and highlights its potential for integration into future diagnostic platforms, including those aimed at neurodegenerative disease research.

## Author contributions

GG: conceptualization; project coordinator; investigation, data curation; writing. ADL: investigation. LFB: data curation, review – editing. PP: conceptualization; investigation, data curation; review – editing; FB: conceptualization; investigation, data curation; review – editing.

## Conflicts of interest

There are no conflicts to declare.

## Supplementary Material

NR-017-D5NR03358F-s001

## Data Availability

All authors confirm that the data supporting the manuscript have been included as part of the supplementary information (SI). Supplementary information is available. See DOI: https://doi.org/10.1039/d5nr03358f.
