# Supplementary material for: Clay-dye fluorescent hybrid material for the detection of neurotransmitter secretion from cells
Source: Nanoscale. 2025 Oct 28;17(46):26942–51. doi: 10.1039/d5nr03358f (PMC12604653; doi:10.1039/d5nr03358f)
Supplement: NR-017-D5NR03358F-s001 [file NR-017-D5NR03358F-s001.pdf]

Supplementary Information (SI) for Nanoscale.
This journal is © The Royal Society of Chemistry 2025

Supporting Information

Clay-Dye Fluorescent Hybrid Material for the Detection of Neurotransmitter Secretion from Cells

Giorgia Giovannini[ab], Ada De Luigi[a], Luciano F. Boesel[b], Pierre Picchetti[c*], and Frank Biedermann [c*]*

[a] Department of Biochemistry and Molecular Pharmacology, Mario Negri Institute for Pharmacological Research IRCCS, Via Mario Negri 2, Milano, Italy

[b] Empa, Swiss Federal Laboratories for Materials Science and Technology, Laboratory for Biomimetic Membranes and Textiles, Lerchenfeldstrasse 5, St. Gallen 9014, Switzerland

[c] Institute of Nanotechnology (INT) Karlsruhe Institute of Technology (KIT), Kaiserstrasse 12, 76131 Karlsruhe, Germany

*Correspondence should be directed to:

giorgia.giovannini@marionegri.it, pierre.picchetti@kit.edu and, frank.biedermann@kit.edu.

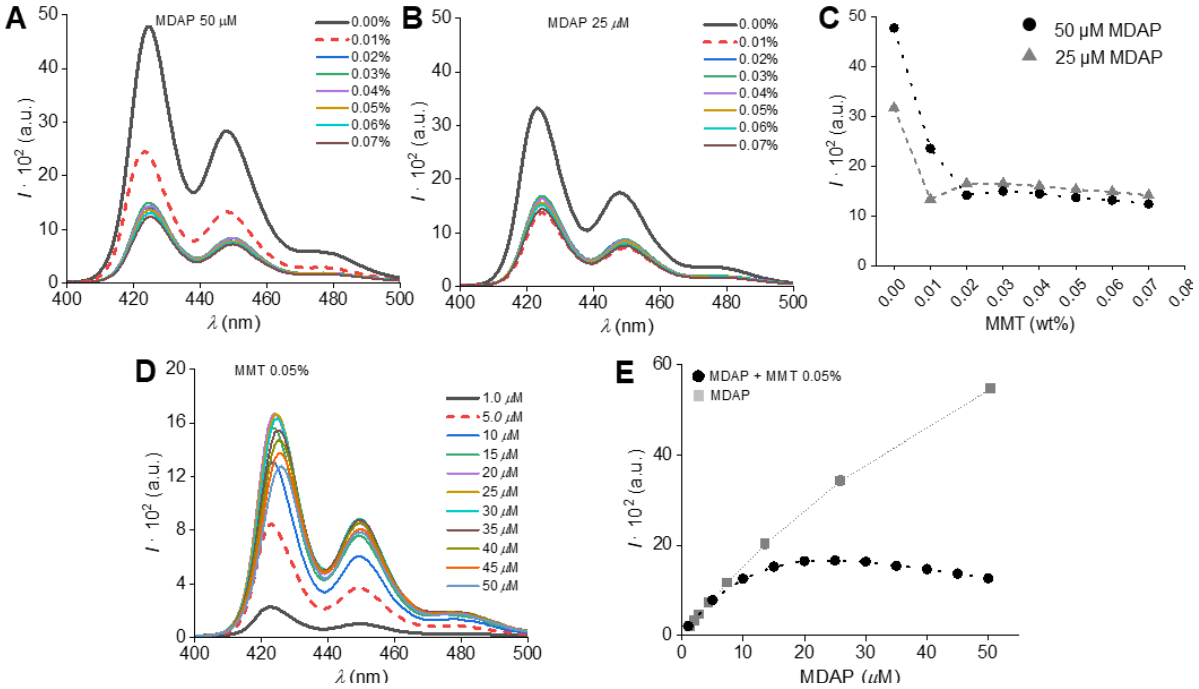

Figure S1. Titration of 50 µM (A) and 25 µM (B) MDAP with MMT at different concentrations (0-0.07 %). C) Comparison between the two MDAP concentrations, highlighting the saturation point of the quenching phenomenon. D) Titration of 0.05 % MMT with different concentrations of MDAP (0-50 µM). The quenching effect was visible at 10 µM of MDAP, reaching a maximum at 50 µM (E).

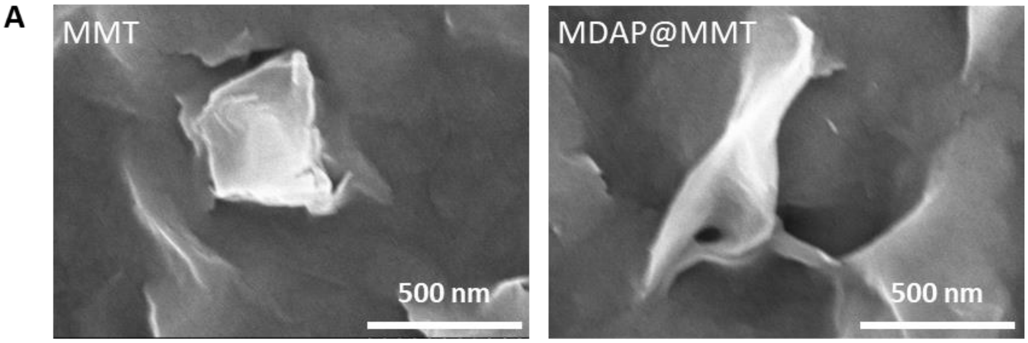

B

	MMT	MDAP@MMT
ZP [mV]	-22,57 ± 0,42	-31,37 ± 1,68

Figure S2. A) Morphological characterization of pristine MMT and MDAP@MMT (50 µM MDAP to 0.05% MMT) via SEM indicating that the particles maintain their typical layered structure. B) Zeta potential measured for pristine MMT (0.05 %) and of the final probe MDAP@MMT (50 µM and 0.05 % respectively) showing a slight decrease of MMT's surface charge upon MDAP adsorption. Data represent mean values ± standard deviation (n = 3).

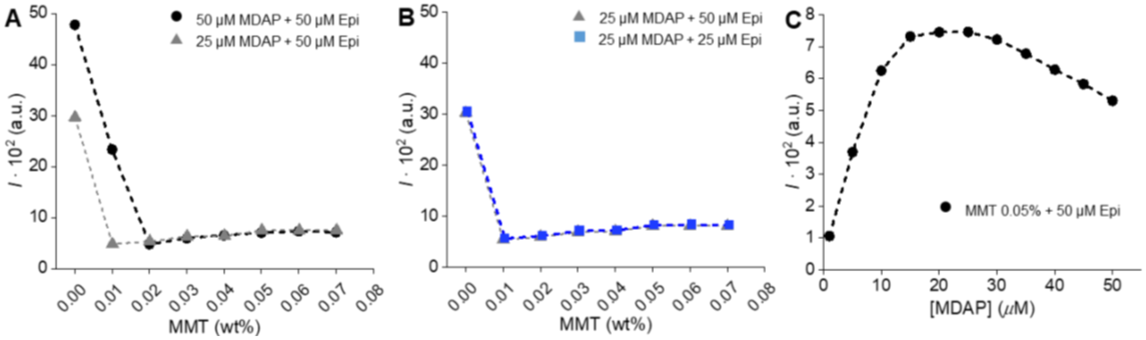

Figure S3. Evaluation of sensor performance by varying the MDAP/MMT ratio. Panels A–C demonstrate that the MDAP concentration primarily governs the saturation behavior of the sensor.

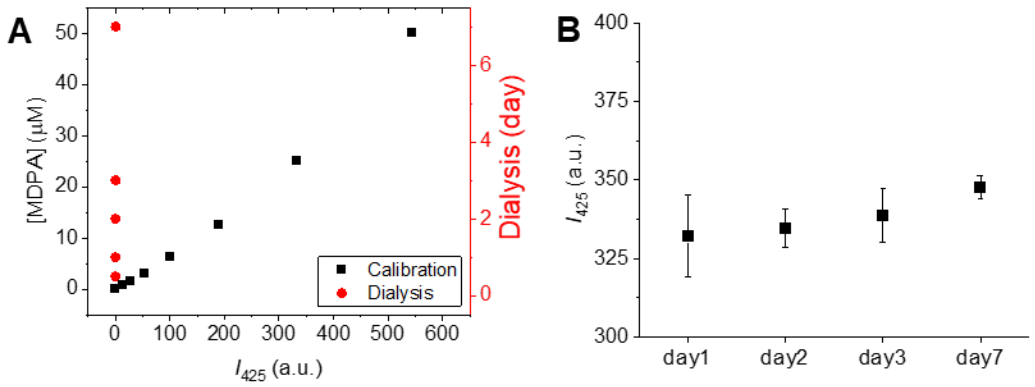

Figure S4. A) After 7 days of dialysis, no traces of MDAP were detected in the solution (red dots) as quantified with the calibration curve of MDAP (black squares). B) The signal of the MDAP@MMT remain stable during the 7-days dialysis. Values are reported as average values (n=3), ± sd.

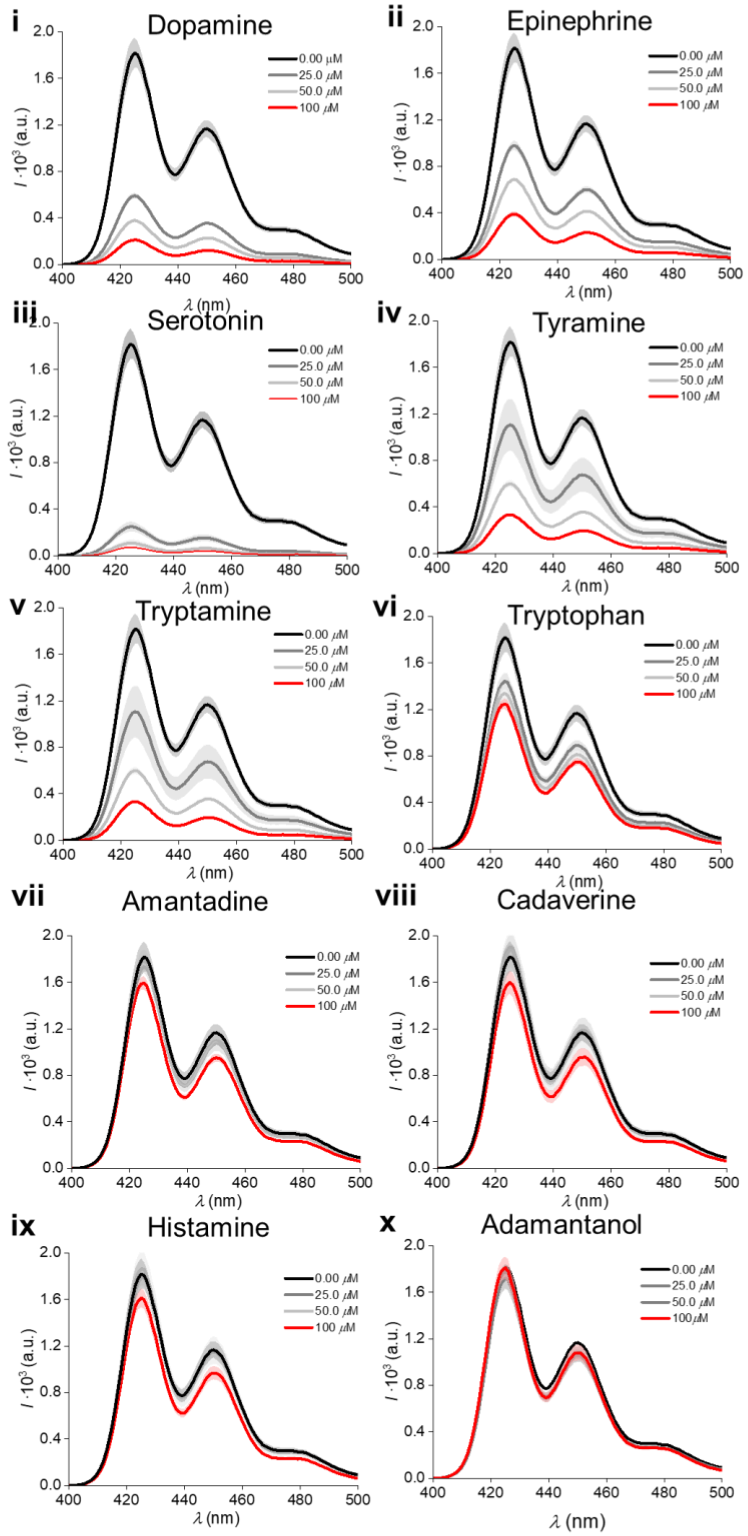

Figure S5. Attenuation of MDAP@MMT fluorescence upon addition of selected neurotransmitters, biosynthetic precursors, and other neuroactive metabolites.

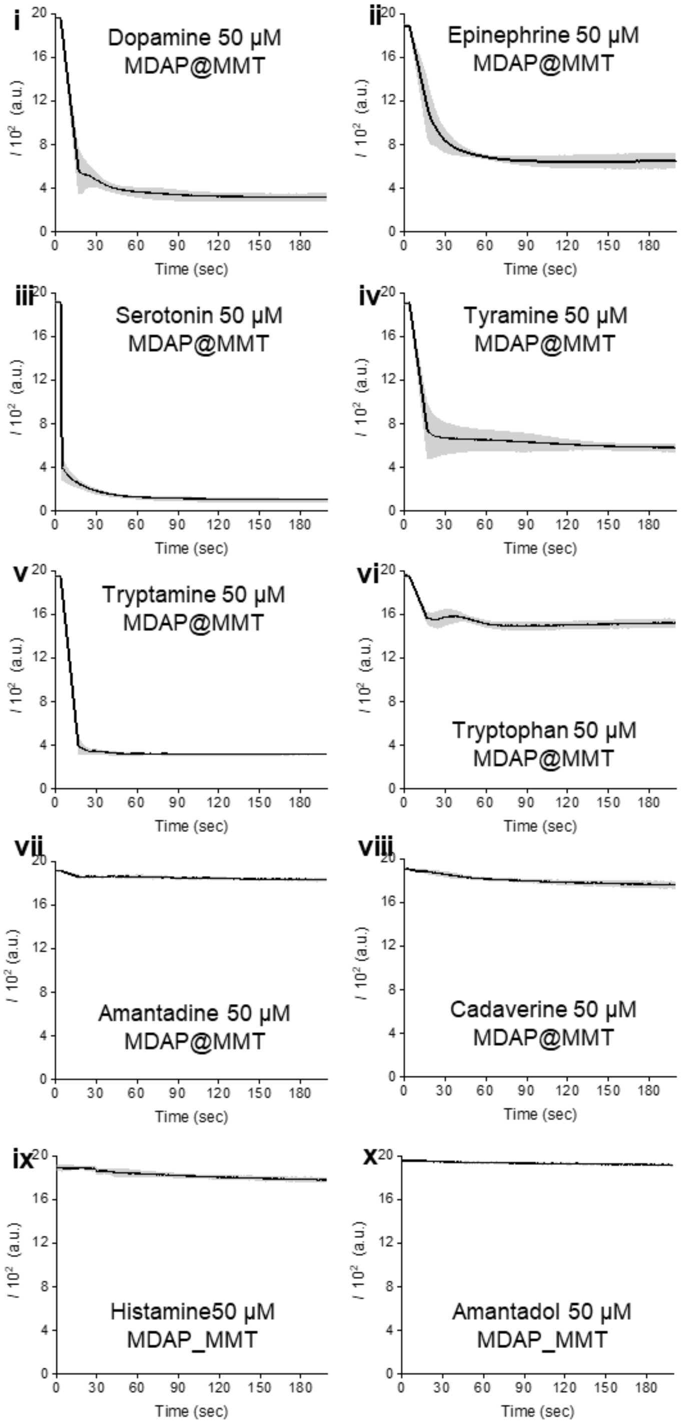

Figure S6.Rapid quenching kinetics of the MDAP@MMT probe upon analyte addition: equilibrium fluorescence is reached within 60 s.

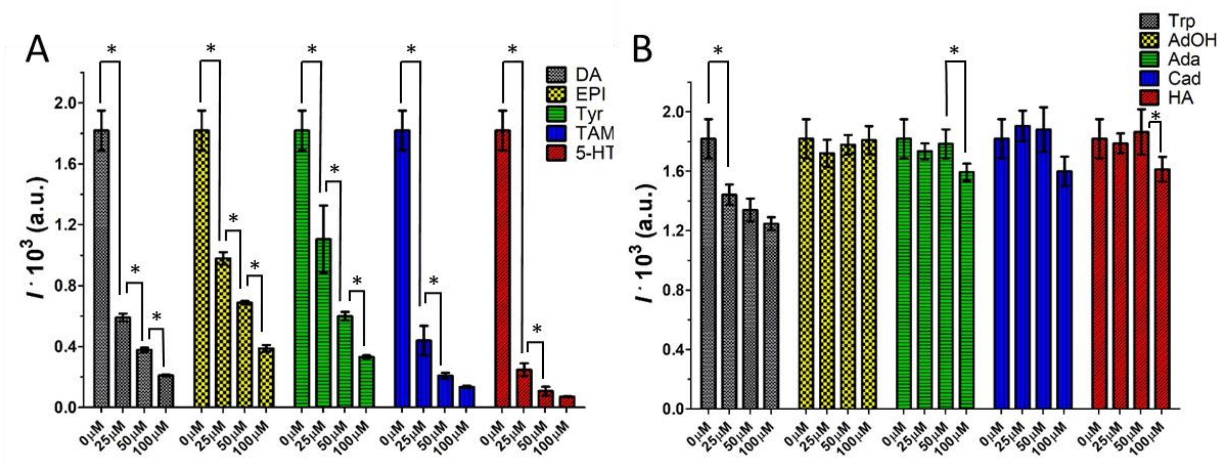

Figure S7: Statistical analysis of responsiveness of MDAP@MMT for different NTs. Data are reported as mean ± sd (n = 3). * Sample means are statistically different (ANOVA One-way, n = 3, α 0.05, post hoc Tukey's test).

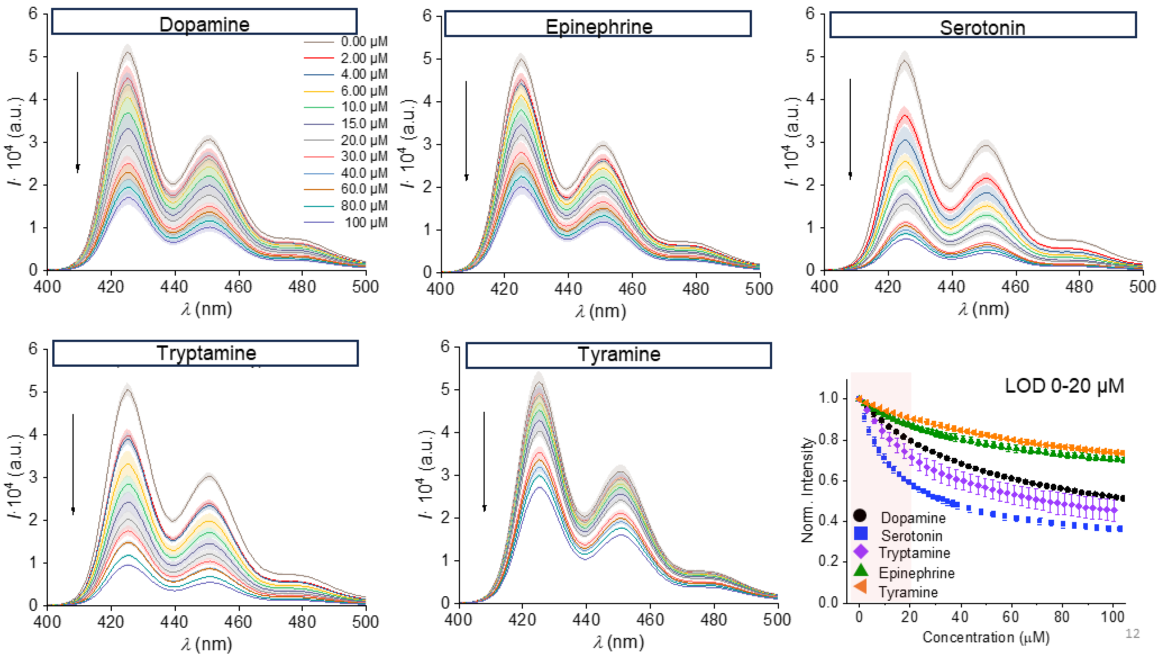

Figure S8. Analytical performance of MDAP@MMT toward catecholamine-type NTs. Limits of detection (μM): dopamine 11.0, serotonin 2.80, epinephrine 31.0, L-tryptamine 8.35, L-tyramine 19.2.

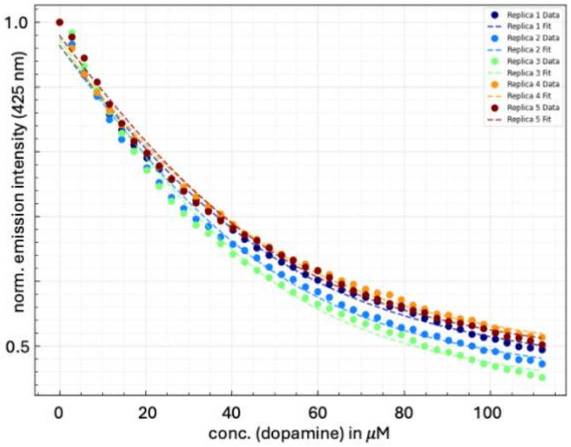

Figure S9: Dopamine titration of MDAP@MMT (0.05 wt % MMT, 50 µM MDAP, 25 °. Normalized emission at 425 nm vs DA concentration for five automated replicas (symbols) and their 1:1 fits (lines). A 1:1 direct-binding model (DBA) was fitted to five independent titrations. For each replicate the parameters Ka, Io, IMDAP@MMT and I(DA•MDAP)@MMT were optimized with RMSE and R^2. The average fit result is Ka = (8.3 ± 1.1) × 104 M^{-1}. These values confirm a high-affinity 1:1 interaction between dopamine and the MDAP·MMT suspension in aqueous media.

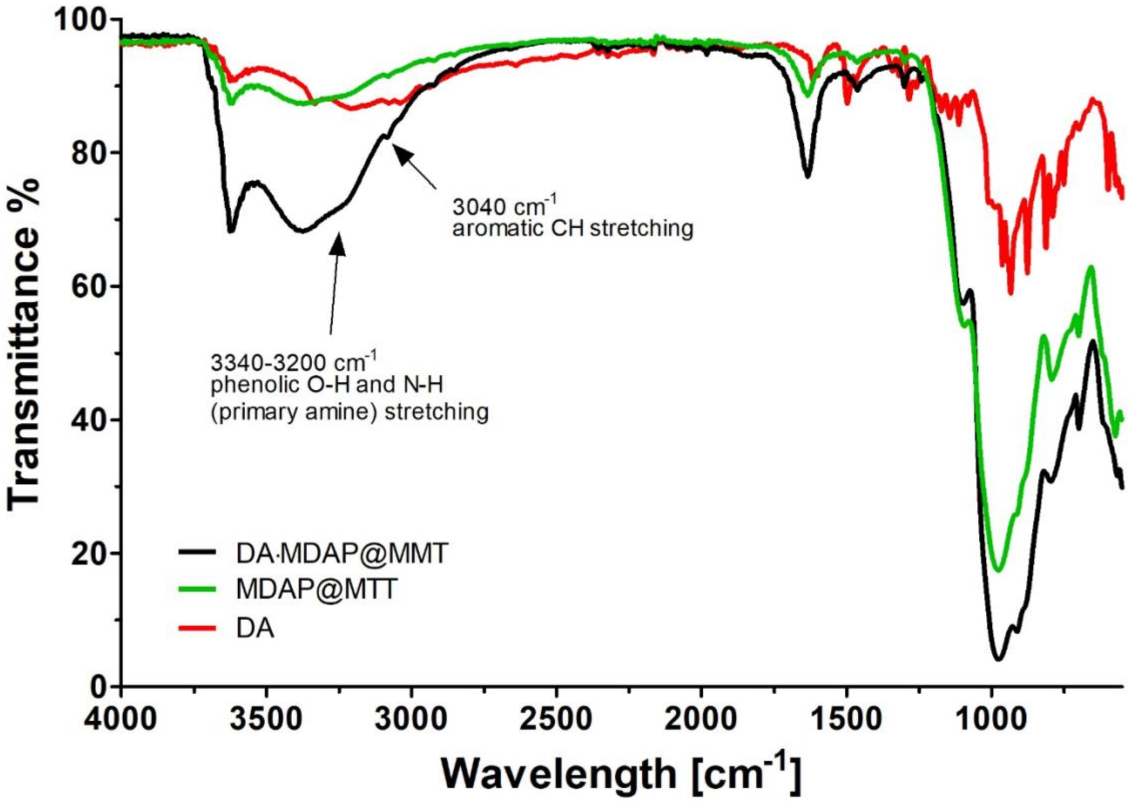

Figure S10: Figure S 8: FT-IT analysis confirming the adsorption of NTs on the probe. After treatment with 100 µM of DA, the powder isolated by centrifugation and dried showed the peaks typically assigned to the N-H, O-H, and C-H stretching of DA, which are not present in the spectra of MDAP@MMT.

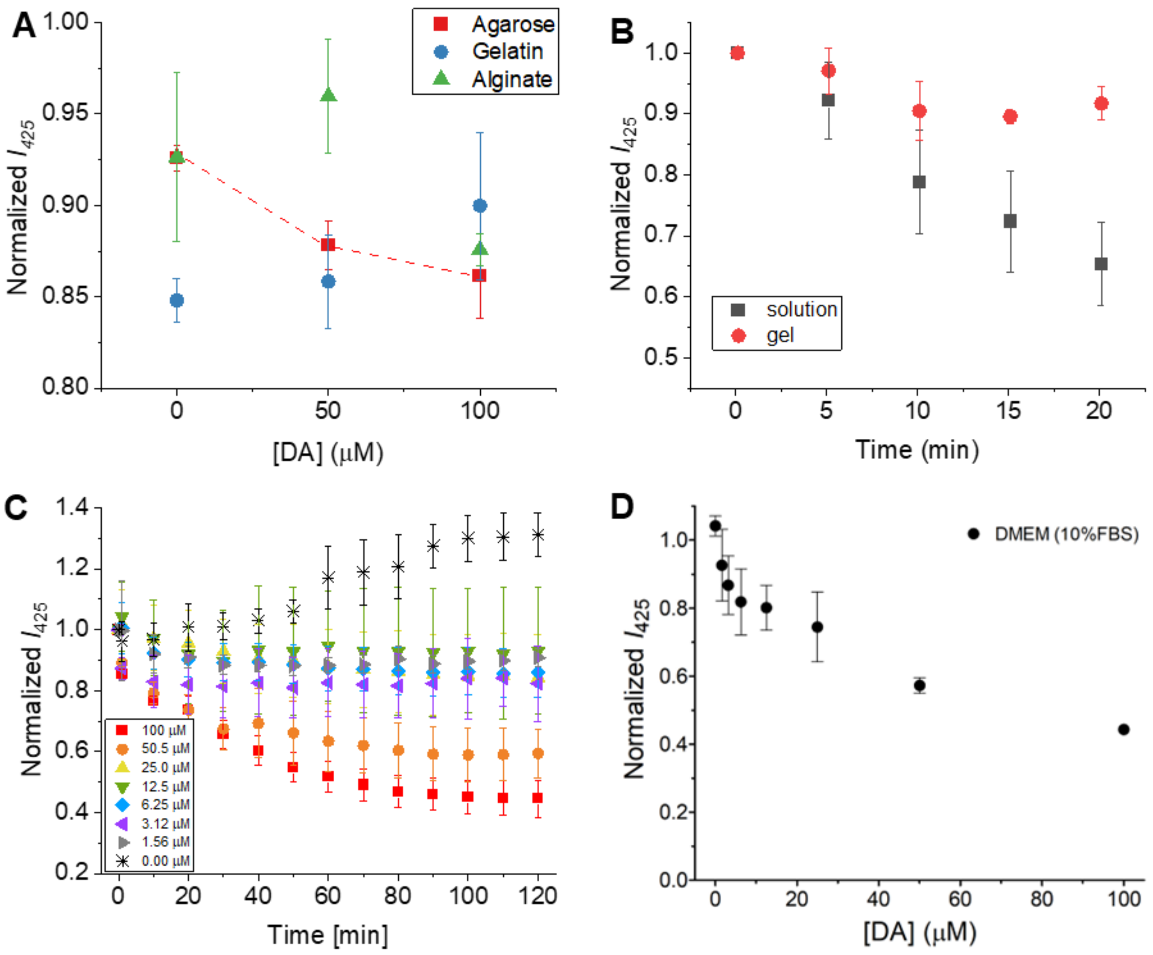

Figure S11. A) Sensing activity of MDAP@MMT in the presence of protein is retained only when the probe is immobilized in agarose hydrogel. B) Signal stability over time of free MDAP@MMT (black squares) compared to agarose-embedded MDAP@MMT (red circles) in cell culture medium (DMEM) supplemented with 10% fetal bovine serum (FBS). C) Fluorescence response of agarose-embedded MDAP@MMT to increasing concentrations of dopamine, in DMEM + 10% FBS. D) Responsiveness of agarose-embedded MDAP@MMT toward dopamine (0-100 µM) in DMEM + 10% FBS (no phenol red). Data are plotted as mean ± standard deviation (n = 3).

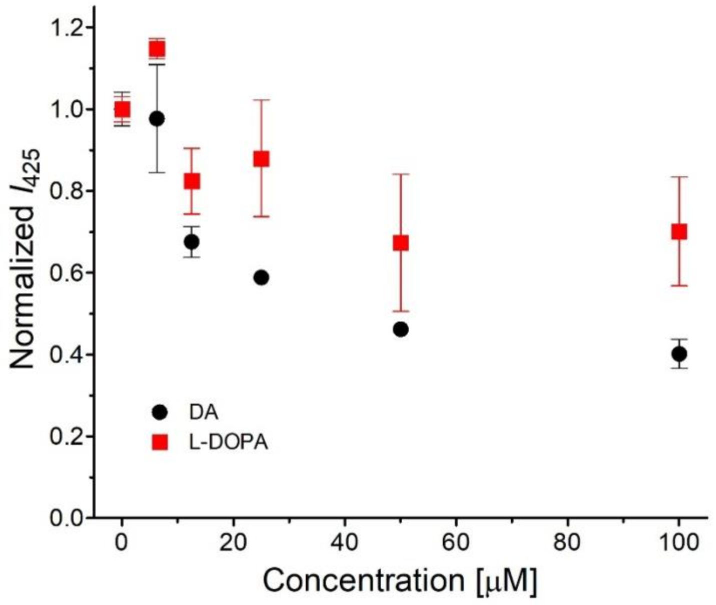

Figure S 12: Comparison of the MDAP@MMT's signal variation when treated with DA or L-DOPA. The unstable adsorption of L-DOPA that could explain the fluctuation of signal intensity was attributed to its zwitterionic form.

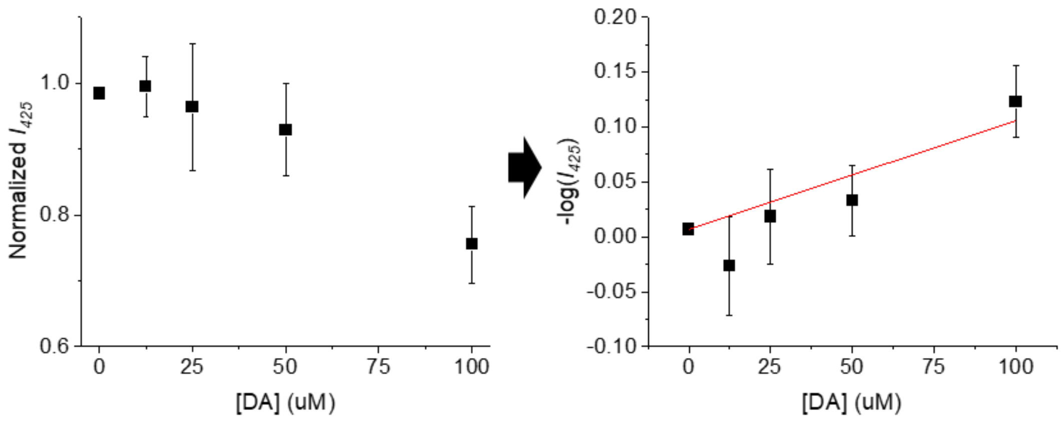

Figure S13. Calibration curve for DA in HBSS (R^2 = 0.86, slope = 1.4) employed for quantifying dopamine release from dopaminergic cells after CU treatment. Values are mean ± SD (n = 3).

Table S1: Summary of representative DA detection systems, highlighting their key features and corresponding limits of detection (LOD).

System	***LOD (for DA)***	***Sensing Conditions***	***Key Materials / Probes***	***Reference***
Aptamer-functionalized graphene FET (electrochemical transistor)	1 aM (10^{-18} M)	Artificial cerebrospinal fluid (aCSF); DA-spiked brain homogenate; pH ~7.4	Graphene multitransistor array functionalized with dopamine-binding aptamer	Abrantes M. et al.[1]
DNA-origami SERS sensor (plasmonic nanostar dimer)	0.225 fM (2.25×10^{-16} M)	Aqueous buffer; dried on SERS substrate	DNA origami-templated Au@Ag nanostar dimers	Kaur V. et al.[2]
Spread-spectrum SERS (label-free)	1.9 aM (1.9×10^{-18} M)	Saline solution (0.9% NaCl)	Au nanoisland SERS substrate with spread-spectrum excitation	Lee W. et al.[3]
Fluorescent Au nanocluster (turn-off)	0.62 nM (PBS) / 0.83 nM (CSF)	PBS buffer (0.5 mM, pH 7.2) and spiked human CSF	BSA-stabilized Au nanoclusters	Govindaraju S. et al.[4]
Fluorescent carbon dots (boronic acid functionalized, turn-on)	0.1 pM (1×10^{-13} M)	Aqueous buffer, neutral pH	B–N co-doped carbon dots with boronic acid and amine groups	Liu X. et al.[5]
Fluorescent polydopamine nanoparticles (turn-on)	40 nM	Alkaline solution (Tris/NaOH), pH > 8	Dopamine self-polymerization to polydopamine nanoparticles	Yildirim A. et al.[6]
Fluorescent carbon dot probe (in situ synthesis)	56.2 nM	Mild aqueous conditions (37 °C); tested in human serum	In situ formation of green fluorescent carbon dots from dopamine and aminosilane (AEATMS)	Tang X. et al.[7]
Colorimetric Au nanoparticle assay	2 nM	Aqueous buffer; tested in artificial CSF	DSP-modified Au nanoparticles with Fe^{3+} crosslinking of catechol groups	Liu L. et al.[8]

References

1. Abrantes, M. *et al.* Ultrasensitive dopamine detection with graphene aptasensor multitransistor arrays. *J. Nanobiotechnology* 20, 495 (2022).

2. Kaur, V., Sharma, M. & Sen, T. DNA Origami-Templated Bimetallic Nanostar Assemblies for Ultra-Sensitive Detection of Dopamine. *Front. Chem.* 9, (2021).

3. Lee, W. *et al.* Spread spectrum SERS allows label-free detection of attomolar neurotransmitters. *Nat. Commun.* 12, 159 (2021).

4. Govindaraju, S., Ankireddy, S. R., Viswanath, B., Kim, J. & Yun, K. Fluorescent Gold Nanoclusters for Selective Detection of Dopamine in Cerebrospinal fluid. *Sci. Rep.* 7, 40298 (2017).

5. Liu, X. *et al.* In situ bifunctionalized carbon dots with boronic acid and amino groups for ultrasensitive dopamine detection. *Anal. Methods* 8, 3236–3241 (2016).

6. Yildirim, A. & Bayindir, M. Turn-on Fluorescent Dopamine Sensing Based on *in Situ* Formation of Visible Light Emitting Polydopamine Nanoparticles. *Anal. Chem.* 86, 5508–5512 (2014).

7. Tang, X.-Y. *et al.* Turn-on fluorescent probe for dopamine detection in solutions and live cells based on in situ formation of aminosilane-functionalized carbon dots. *Anal. Chim. Acta* 1157, 338394 (2021).

8. Liu, L., Li, S., Liu, L., Deng, D. & Xia, N. Simple, sensitive and selective detection of dopamine using dithiobis(succinimidylpropionate)-modified gold nanoparticles as colorimetric probes. *Analyst* 137, 3794 (2012).
